# Creating Interactive Data Dashboards for Evidence Syntheses

**DOI:** 10.1002/cesm.70035

**Published:** 2025-06-25

**Authors:** Leslie A. Perdue, Shaina D. Trevino, Sean Grant, Jennifer S. Lin, Emily E. Tanner‐Smith

**Affiliations:** ^1^ Kaiser Permanente Evidence‐Based Practice Center Portland Oregon USA; ^2^ HEDCO Institute for Evidence‐Based Educational Practice, College of Education University of Oregon Eugene Oregon USA

**Keywords:** dashboard, evidence synthesis, R, shiny, tableau

## Abstract

Systematic review findings are typically disseminated via static outputs, such as scientific manuscripts, which can limit the accessibility and usability for diverse audiences. Interactive data dashboards transform systematic review data into dynamic, user‐friendly visualizations, allowing deeper engagement with evidence synthesis findings. We propose a workflow for creating interactive dashboards to display evidence synthesis results, including three key phases: planning, development, and deployment. Planning involves defining the dashboard objectives and key audiences, selecting the appropriate software (e.g., Tableau or R Shiny) and preparing the data. Development includes designing a user‐friendly interface and specifying interactive elements. Lastly, deployment focuses on making it available to users and utilizing user‐testing. Throughout all phases, we emphasize seeking and incorporating interest‐holder input and aligning dashboards with the intended audience's needs. To demonstrate this workflow, we provide two examples from previous systematic reviews. The first dashboard, created in Tableau, presents findings from a meta‐analysis to support a U.S. Preventive Services Task Force recommendation on lipid disorder screening in children, while the second utilizes R Shiny to display data from a scoping review on the 4‐day school week among K‐12 students in the U.S. Both dashboards incorporate interactive elements to present complex evidence tailored to different interest‐holders, including non‐research audiences. Interactive dashboards can enhance the utility of evidence syntheses by providing a user‐friendly tool for interest‐holders to explore data relevant to their specific needs. This workflow can be adapted to create interactive dashboards in flexible formats to increase the use and accessibility of systematic review findings.

## Summary

- Traditional systematic review data are complex, and outputs are often static and primarily designed for a research audience, limiting their accessibility and utility for diverse audiences.
- This paper introduces a workflow or creating interactive data dashboards specifically for systematic reviews, with its application demonstrated through two real‐world examples from previous reviews in healthcare and education.
- Interactive dashboards transform how systematic review findings are shared, enhancing accessibility, engagement, and practical application across diverse audiences.
- This workflow provides readers with actionable guidance to make systematic review findings more relevant to their intended audiences, helping to reduce the research to practice gap.

## 1 Introduction

A vital output of an evidence synthesis is the data set compiling the information collected from included studies [1]. Findings from analyses of evidence synthesis datasets are typically communicated in manuscripts, tables, and figures. Given evidence syntheses often yield large amounts of data, traditional dissemination approaches have important limitations [2, 3]. For example, manuscript text, tables, and figures are nearly always static and most often not designed in consultation with the intended users of evidence synthesis findings. In addition, evidence synthesis datasets are not always shared along with manuscripts, preventing others from reanalyzing the data to answer questions and provide analyses not contained in manuscripts [4]. Consequently, the use of findings largely rests on the information presented and format for presenting them chosen by the evidence synthesis team, with no ability for the end‐user to tailor the information presented and how it's visualized.

Interactive data dashboards offer a useful tool to convey information from evidence syntheses beyond what can be accomplished with text and static output [5]. Also known as interactive web applications, interactive data dashboards are online tools that transform static datasets into dynamic, user‐friendly visualizations, enabling users to explore and interact with research findings tailored to their needs [6]. Interactivity not only enhances the user experience but also facilitates deeper engagement with evidence synthesis results. Another major advantage of interactive data dashboards is their flexibility in displaying and organizing scientific findings. They allow systematic reviewers to present any type of systematic review data or results in various formats that can be tailored to different audiences. Additionally, dashboards support a wide range of data visualizations, including interactive charts, tables, plots, pictures, and dynamically generated text summaries. Incorporating different types of visualizations based on the data presented makes it easier for users to locate and understand the information that is most relevant to them [7]. Lastly, platforms for interactive data dashboards also have functionality that allows users to download the underlying data set and upload their own data set. By offering interest‐holders the ability to explore data specific to their needs and contexts, interactive dashboards can make evidence syntheses more accessible and actionable [8].

A recent scoping systematic review documented an increased use of interactive visualizations in many areas of health, particularly visualizations embedded within health care organizations [9]. However, interactive data dashboards have not been widely utilized to display results from systematic reviews, although their use has been previously explored [10]. This manuscript provides a tutorial on creating an interactive data dashboard for an evidence synthesis using two real‐world examples.

## 2 Workflow for Creating an Interactive Data Dashboard in an Evidence Synthesis

We provide a generic workflow for generating data to use and for creating the dashboard. Although our examples use Tableau and R Shiny, we have framed this workflow following common steps of an evidence synthesis to try to be as applicable to any interactive data dashboard software. That said, this paper assumes working knowledge of Tableau, R Shiny, and best practices in evidence synthesis. This first stage involves defining the scope of the dashboard, determining the audience for the dashboard, and preparing the data for the dashboard. Items to consider include the audience and objective of the dashboard, selecting the dashboard software, creating standardized forms for data collection that facilitate relational databases, and preparing the data for import into the dashboard software. For example, when developing the review protocol and data collection tool, be intentional in the data you will need to also use for the dashboard (e.g., variable names, missing data, formatting). This second stage involves designing and building the dashboard. Items to consider include importing the data, defining the interactive elements, and the design and layout of the user interface, including the visualizations, filters, and information to present. This third stage involves deploying the dashboard, user‐testing, and a commitment to continuous quality improvement.

## 3 Worked Examples

Two recent systematic reviews were used to create interactive data dashboards. Specifically, one review was used to support a recommendation statement made by the U.S. Preventive Services Task Force (USPSTF) related to screening children for lipid disorders (Example 1). This systematic review synthesized evidence on screening and treatment for lipid disorders identified in children [11, 12]. The second review was a scoping review to examine the extent of the empirical research evidence on the 4‐day school week in primary and secondary schools in the United States (Example 2) [13].

### 3.1 Example 1: Treatment for Child Lipid Disorders

The interactive dashboard for the systematic review of treatment for child lipid disorders was created to assist the systematic review team and guideline panel end‐users as they interrogated the evidence. The end‐users were all clinicians and researchers familiar with interpreting results from systematic reviews. The standard products generated by the systematic review team for the USPSTF include a detailed evidence report along with an oral presentation and an accompanying slide set. While these products are useful as reference materials and for presentation purposes, neither provide a summary of the evidence in a condensed format. Further, the standard products make it difficult for the user to focus on the evidence for which they are most interested. The objective of this interactive dashboard was to provide the systematic reviewers and end‐users with a condensed snapshot of the evidence on treatment for child lipid disorders. A version of this interactive dashboard is available publicly here: https://www.ahrq.gov/data/visualizations/treatment-fh-mfd.html.

#### 3.1.1 Planning and Preparation

As part of our usual methods for conducting systematic reviews, we used DistillerSR to create standardized forms for data collection. We used a hierarchical data structure that began with the study‐level characteristics at baseline (e.g., age, sex, race/ethnicity, lipid levels) for our first level of data. The second level of data included information about the intervention and control arms, such as intervention category (pharmacologic, supplements, behavioral), medication dose, and description of any behavioral components. The third level of data captured the outcome details as well as the continuous or dichotomous data at each timepoint (e.g., mean LDL at baseline and 6 months of follow‐up). Level 1 data could only be entered once for each study, but both level 2 and level 3 data allowed multiple entries per study to account for several intervention arms and multiple reported outcomes and timepoints. In this project, the study characteristics (Level 1) were only entered for the total randomized sample (intervention and control arms combined). The control group description (Level 2) and results (Level 3) were entered in the same forms as the intervention group, resulting in one row with both the control and intervention group data at a timepoint (rather than a row for each). While this design does result in control group information being entered multiple times if a study has more than one intervention group, the exported data file is structured as needed for analysis (meta‐analyses in Stata), Tableau, and our evidence reports. The relational data were exported from DistillerSR as a flattened Excel file; the export from DistillerSR duplicates single forms (Level 1 and 2) when repeating forms (Level 2 or 3) are present. Appendix Figure S1 displays selected screen shots of the forms in DistillerSR into which data were entered, and Table 1 shows an example of how the data were structured in the resulting flattened file.

**Table 1:** Example of a flattened^a^ hierarchical data table exported from DistillerSR.

LEVEL 1							LEVEL 2				LEVEL 3					
Refid	Author Year	Design	Condition	Totaln	AgeMean	FemalePct	Arm	Medication	Dose	Control	Outcome	Time	IGn	CGn	IGmean	CGmean
250	Braamskamp, 2015	RCT	FH	106	10.6	54.7	IG1	Pitavastatin	4 mg/d	Placebo	LDL	12	24	27	144.4	239.2
250	Braamskamp, 2015	RCT	FH	106	10.6	54.7	IG1	Pitavastatin	4 mg/d	Placebo	LDL	0	24	27	240.7	240.5
250	Braamskamp, 2015	RCT	FH	106	10.6	54.7	IG2	Pitavastatin	2 mg/d	Placebo	LDL	12	26	27	156.8	239.2
250	Braamskamp, 2015	RCT	FH	106	10.6	54.7	IG2	Pitavastatin	2 mg/d	Placebo	LDL	0	26	27	223.1	240.5
250	Braamskamp, 2015	RCT	FH	106	10.6	54.7	IG3	Pitavastatin	1 mg/d	Placebo	LDL	12	26	27	231.4	239.2
250	Braamskamp, 2015	RCT	FH	106	10.6	54.7	IG3	Pitavastatin	1 mg/d	Placebo	LDL	0	26	27	176.3	240.5

a DistillerSR stores data in a hierarchical data structure. When this file is exported from Distiller to Excel, the one‐to‐many relationship between the data in Level 1 and subsequent levels is “flattened” and the data from Level 1 are repeated in each row. In this example, the author, study design, condition, study size (total n), mean age and percent female were all repeated when multiple intervention arms were added for Level 2 (IG1 and IG2).

Once data were collected into DistillerSR, we imported the flattened file into Stata to standardize the outcome data. The structure of the data file was not changed, but some data cleaning, unit conversion, and specific calculations were required to standardize the data between studies. The majority of data for this topic were continuous measures, so a between group difference in change and associated confidence interval was calculated for each lipid outcome (LDL‐cholesterol, HDL‐cholesterol, non‐HDL‐cholesterol, total cholesterol, and triglycerides) in units standard for US clinical practice (mg/dL).

#### 3.1.2 Development

The evidence synthesis data were imported into Tableau to generate the interactive dashboard. The flattened, hierarchical format of the data as exported from DistillerSR was appropriate for Tableau. Tableau automatically determined the data type (e.g., ‘measure’ or numeric, ‘dimension’ or string) based on what was found in each data column, but this was adjusted in Tableau as needed. Separate visualizations were created for a variety of measures: number of studies and participants, mean and range of participant age, baseline lipid levels for participants, and a forest plot of the mean difference in change for the outcome selected. The structure of the data (repeating data for measures in Level 1) meant that each time a numeric variable was used from Level 1, the measure needed to be adjusted to an “average” instead of the “sum” default in Tableau. For example, if a study reported a mean age of 10 years but had 6 rows of data in the file, the default in Tableau would be to display a sum of 60.

Further, as Tableau does not have a built‐in option for a forest plot, minimal calculations were made in Tableau to create the forest plot. To generate a forest plot, the point estimate (this ‘measure’ was placed in the ‘columns’) was first plotted against the study author (this ‘dimension’ was placed in the ‘rows’). The preferred shape was selected for the point estimate of the mean difference in change. The measure for the lower confidence interval measure was also placed on the same plot and the display converted to a Gantt Bar for the lower confidence interval only. A dual axis was selected, the axes were synchronized, and the top header was hidden. The span of the confidence intervals was calculated (upper CI minus lower CI) and this was used for the “size” of the Gantt Bar. This resulted in a typical forest plot displaying point estimates and confidence intervals. Additional dimensions were added to columns in the forest plot, such as the baseline and follow‐up values for the selected outcome. Once individual visualizations were generated, each was added to the dashboard to display the effects of treatment in children with elevated lipids. Filtering options for intervention group and outcome were added. The use of color and shapes denoted the condition recruited for each study (familial hypercholesterolemia, multifactorial dyslipidemia, or both). The tooltip option was utilized to display detailed data.

#### 3.1.3 Deployment

The draft interactive dashboard was first shared internally with the systematic review team. During this stage, adjustments to the dashboard were made as needed to correct for any errors made during the earlier stages (e.g., data abstraction errors made in DistillerSR, data analysis errors in Stata, errors made when generating the individual visualizations in Tableau). The interactive data dashboard facilitated the identification of errors made in these earlier stages. Further adjustments were made based on the preferences of the systematic review team, such as the data selected to display and the specific visualizations they preferred.

The systematic review team accessed the interactive data dashboard via Tableau Reader, a free desktop application that can be used to view a packaged workbook created in Tableau. As the systematic review was not yet publicly available, the end‐users accessed the data dashboard online through a secure Tableau Server. Before the data dashboard was available to a public audience and to meet accessibility requirements, the layout was adjusted for smaller displays, and we ensured the tab order followed the order of the individual visualizations in the dashboard. Based on additional user feedback, some visualizations were removed from the data dashboard to streamline the design. Further adjustments were made to the shape and color selections of the individual visualizations to clarify the condition designation.

### 3.2 Example 2: Four‐Day School Week

**Figure 1:**
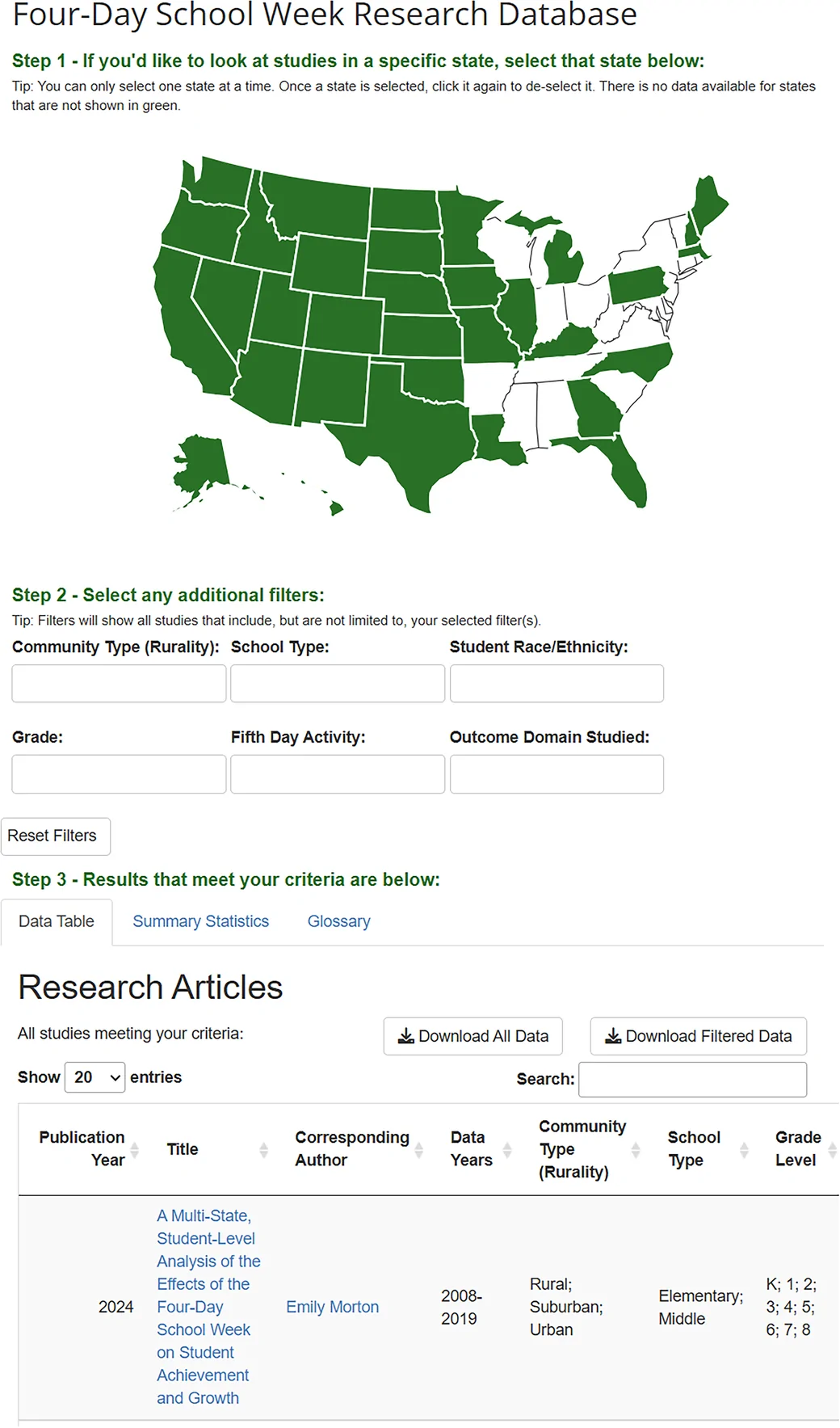
Interactive dashboard displaying the data on the 4‐day school week in the U.S. * The following columns were removed from this screenshot for readability: student race and/or ethnicity, state, fifth day activities, outcome domain, equity domain, and implementation domain.

The interactive dashboard for the scoping review on the 4‐day school week in the United States was created using R and the Shiny package [14]. This dashboard was specifically designed to help non‐research audiences access and explore data from empirical studies on the 4‐day school week (Figure 1). Since these data came from a scoping review, rather than a meta‐analysis, there is no information on policy effectiveness but instead there is extensive descriptive information available about each empirical study that reported on the 4‐day school week in the United States. Our goal was to present this descriptive information extracted from all studies in a user‐friendly way that allowed interest‐holders to find information that is relevant to their specific educational context. The 4‐day school week dashboard is publicly available online: https://hedcoinstitute.uoregon.edu/dashboards/4-day-school-week-research-database.

#### 3.2.1 Planning and Preparation

Our first step in creating the 4‐day school week data dashboard was to define the audience and objective. We aimed to develop an interactive tool that allowed users to explore data extracted from the scoping review and filter the data by various study characteristics (e.g., school type, student race/ethnicity, location). Since our target audience was non‐researchers, the goal was to make this dashboard as user‐friendly as possible. To achieve this, we planned to include clear instructions on how to use the dashboard, minimize the amount of scientific jargon, and create data filters so users can search for information relevant to their specific context. We chose R Shiny for its highly customizable code‐based interface, flexibility for making changes or updates, and seamless integration with R for data transformation and analysis. It is also open source, which increases the reproducibility and transparency of our work and allows users to freely access and use the code and data.

Next, we prepared the data for use in the dashboard. Data were exported as a series of flat files from DistillerSR, the systematic review management software used for the scoping review, then imported to R for pre‐processing. Pre‐processing included merging all files together so that all study characteristics and reference information were in one data frame and correcting the formatting of text responses. As our objective was to present an uncomplicated data table for users to explore in the dashboard, we restructured the data to be more user‐friendly by including a subset of variables that would be most relevant to interest‐holders, converting responses to be more descriptive, and grouping select‐all variables into one column with multiple answers separated by a semi‐colon. We then exported a single flat file for use in the dashboard. After pre‐processing, we imported the single flat file into R for building the dashboard script. When we decided on additional data transformations to make after pre‐processing was complete (e.g., aggregating responses, reducing jargon, shortening responses to fit in table, adding hyperlinks), those transformations were made within the dashboard script to denote they were updates to our pre‐registered plan.

#### 3.2.2 Development

The design and layout of the user interface was crucial to making the dashboard intuitive to non‐research audiences. We aimed for a clean layout with easy‐to‐use filters and concise data displays to encourage user interaction and data exploration. The color scheme we chose was meant to compliment the host institution's webpage color scheme, where the dashboard would be published. To increase usability for non‐research audiences, we designed the dashboard with a step‐by‐step walkthrough format that guided users through the process of interacting with the dashboard with clear instructions. To encourage interactivity, we implemented filters where users could select multiple criteria and dynamically update the displayed data. Filters included state, rurality (e.g., urban), school type (e.g., elementary), student race/ethnicity, grade level, type of activity offered on the 5th day (e.g., teacher in‐service), and what outcomes were studied (e.g., student achievement, staff retention), allowing users to tailor information to their needs.

During the design phase, we worked closely with a communications team to ensure our dashboard was suitable for non‐research audiences. Through this collaboration, we decided to include hyperlinks to the full‐text article of each study and links to the webpage of the main author of the study, when available, so users could access additional information. We also decided to include a map of all U.S. states for users to filter data based on state which was more intuitive than a drop‐down filter. For the data table, we aimed to include all study characteristics that were extracted in the scoping review. Variables were presented in order of which variables we believed would be of most interest to education interest‐holders (and drawing on findings from a qualitative interview study that sought this feedback), with the most important variables presented first [15].

After the design phase, we started setting up a new Shiny project in R. This involved installing and loading the required packages (e.g., shiny, tidyverse, DT, plotly), importing the preprocessed data, and creating the basic structure of the Shiny app (i.e., data dashboard). The basic structure of a Shiny app consists of two major sections, the user‐interface (UI) and the server logic, both of which are usually defined within a single R file named "app.R." The UI defines how the users interact with the dashboard and the server logic processes their interactions to generate dynamic outputs that are displayed back in the UI. The UI section is where the layout and appearance of the dashboard are defined, including where text, interactive elements (e.g., filters, inputs), and other components of the dashboard (e.g., tables, images, outputs) are placed, how they are presented, how users will interact with them. To create our UI, we utilized Shiny's “fluidPage()” layout function due to its flexibility for customizing the layout (see Appendix S1). The server logic is where user inputs are processed, calculations are performed, and outputs are dynamically generated so they can be presented in the UI. In our dashboard, we use the server logic to generate the interactive U.S. map and filters based on user selection, display the filtered data in a nicely formatted table, calculate and display summary statistics, and provide buttons for downloading the complete and filtered data (see Appendix S2). All data files and code to create this dashboard can be found on GitHub: https://github.com/HEDCO-Institute/4DSW_Scoping_Review.

#### 3.2.3 Deployment

After defining the UI and server logic, we used the “shinyApp()” function to render our dashboard within R Studio for local testing. During this testing phase, we ensured all features of the dashboard functioned appropriately, including user inputs, interactivity, output displays, and complex and empty filters. Once the dashboard functioned as expected, we deployed it online so users could access it easily without needing knowledge of R. We chose to deploy through shinyapps. io due to its seamless integration with R Shiny. Using the “deployApp()” function from the *rsconnect* package [16], we deploy our dashboard by specifying the file path to the "app.R” file. After deployment, we used the URL to create an HTML iframe to embed the dashboard within our organization's website. Regular maintenance is crucial to ensuring the dashboard is current and functional. When necessary, we fix broken links, update the data as new information is available, and test and resolve additional errors.

Before launching the dashboard on our website, we conducted additional user‐testing with non‐researchers to ensure the dashboard was intuitive and engaging. Feedback from this testing was incorporated to improve the interface, functionality, and clarity of information presented. Based on this feedback, we added the ability to download both the complete and filtered data, made design improvements such as outlining states on the map and bolding instructional text, and resolved multiple errors users were receiving due to multiple complex filters. We also added the glossary section and reduced technical language throughout the dashboard to further improve clarity and useability, and we added a section to present summary statistics on each variable that dynamically updated with filter selections. To further enhance the utility of our dashboard, we included a feedback form on our website for users to fill out with any comments about the dashboard. We actively review any comments and incorporate user feedback whenever possible.

## 4 Discussion

Creating interactive data dashboards from systematic review datasets is feasible, useful, and can be made accessible for both research and non‐research audiences. When generating an interactive dashboard, an important consideration is the intended audience(s), as the needs and preferences of the intended audience should inform the level of detail presented [17]. Some end‐users may not find a detailed and complex dashboard as helpful as the systematic reviewers themselves might find it; a more digestible version with user‐friendly features and higher‐level synthesis may be appropriate for practitioners, policymakers, community members, or other interest‐holders. Ideally, end‐user needs should guide the type of information that is presented in data dashboards aimed at different audiences. For example, the dashboard made for the treatment of lipid disorders (Example 1) was designed for a guideline panel familiar with interrogating systematic review data. However, the 4‐day school week dashboard (Example 2) was created to make empirical data accessible to non‐research audiences. In both examples, however, we developed these dashboards by first defining the scope, preparing the data, and designing the user experience. We built user‐friendly layouts and a deployed responsive and interactive dashboard experience. And after deploying the dashboard online, we conducted extensive user‐testing and continue to provide regular maintenance for ongoing improvement.

While interactive data dashboards are useful for end‐users in interpreting results from our systematic reviews, they can be just as useful for the review team. An unexpected benefit in adding interactive data dashboards to our review process was the ability to easily identify errors in the data. Although our data is abstracted by one reviewer and checked by another, human errors are inevitable. Other researchers have documented that while errors decrease with two reviewers extracting data, some errors in the data persist [18]. We often find errors at our data visualization stage—such as a missing negative sign, missing decimal, or incorrect number of participants—because they show as extreme outliers or an unlikely or impossible result. Visual displays of data have also enhanced our interpretation of the results, allowing us to identify patterns or trends in the data that are not easily identifiable from tables. This is particularly useful when we are unable to pool results in a meta‐analysis.

The software we selected to create these dashboards—Tableau and R Shiny—both have their own unique drawbacks. Some users may find Tableau licensing to be cost prohibitive. While Tableau has a free version available (Tableau Public) with many of the same core features as the paid versions, it does limit some features, such as the type of data sources to which it can connect. Importantly, the free version is meant to share visualizations online with the data visualization community; those who need to keep their visualizations confidential (such as visualizations with patient or proprietary data) are not the intended users of Tableau Public. Some users may need to find alternative software to build their visualizations, such as Microsoft Power BI (paid and free versions available), Plotly Dash Enterprise (open source), or Apache Superset (open source).

Although R Shiny is open source, building and maintaining a Shiny dashboard requires programming knowledge in R, which can pose barriers to some users. Shiny dashboards can also face performance issues with large datasets or complex computational elements. Additionally, deploying Shiny dashboards—especially for public use—can be complex. Hosting options such as shinyapps. io may involve additional costs and may also require technical expertise. The free tier of shinyapps. io includes limitations such as cold‐start delays, monthly usage caps, limited concurrent‐user capacity, and automatic time‐outs. More robust open‐source options include self‐hosted Shiny Server or Docker‐based deployment with ShinyProxy, though these require additional technical expertise. Paid options such as higher‐tier plans of shinyapps. io or cloud platforms like AWS or Axure may offer greater reliability and support.

While both of our dashboards use only aggregate data from evidence syntheses, researchers publishing dashboards online should consider data privacy and confidentiality, particularly when working with participant‐level, proprietary information, or sensitive data. In addition, the dashboards presented here are not intended to replace formal tools used to assess risk of bias or certainty of evidence (e.g., GRADE tables), but rather to complement them by allowing visual exploration of information collected as part of these evidence syntheses. The decision to not include these assessments in our dashboards was based on the context of each review. In the lipid disorder review, strength of evidence assessments were summarized separately in tables prepared for the panel. While the strength of evidence or certainty was not provided in the dashboard, other relevant characteristics were displayed to help the viewer determine the robustness and applicability of the evidence. For the 4‐day school week review, certainty ratings were not applicable due to its scoping review design. Therefore, a potential limitation of our dashboards is that the simplicity and lack of evidence quality indicators may imply all results are equally robust. When relevant, dashboards can incorporate additional elements, such as risk of bias or GRADE ratings, to help users better interpret and use findings (e.g., see the MetaPsy online application: https://www.metapsy.org/).

The additional effort required to create interactive dashboards can vary considerably depending on factors such as the complexity of the dashboard, the structure of the data, and the experience of the developer. In our experience, when a team member is already familiar with the data structure and review topic, dashboard development can be integrated efficiently into the evidence synthesis workflow. We estimate that, depending on the data science expertise of the project team, building a dashboard would require about 40–80 h of additional work for planning, development, and deployment. This estimate may be lower for teams with prior experience creating dashboards or those with reusable dashboard templates, and they can be scaled down depending on the level of information and interactivity needed. The time requirement may be higher for those who are attempting to create a dashboard for the first time and are unfamiliar with the software or their data set.

## 5 Conclusion

Interactive dashboards provide a powerful solution for presenting systematic review findings in an accessible and actionable format, enabling deeper engagement with complex data. By facilitating interactivity, user‐driven exploration, and tailored results, dashboards are a flexible tool that can be used to facilitate broader dissemination of evidence to both technical and nontechnical audiences. By allowing end‐users to display and explore scientific findings most relevant to their context, interactive dashboards may ultimately assist in the implementation of systematic review findings in applied practice and policy contexts.

## Author Contributions

**Leslie A. Perdue:** conceptualization, visualization, writing – review and editing, writing – original draft, methodology. **Shaina D. Trevino:** conceptualization, visualization, writing – review and editing, writing – original draft, methodology. **Sean Grant:** writing – original draft, writing – review and editing, conceptualization, methodology. **Jennifer S. Lin:** writing – review and editing, conceptualization, funding acquisition. **Emily E. Tanner‐Smith:** writing – review and editing, conceptualization, funding acquisition.

## Conflicts of Interest

The authors declare no conflicts of interest.

## Supporting information

Evidence Synthesis Dashboards appendix.

## Data Availability

Data sharing is not applicable to this article as no new data were created or analyzed in this study.
