# Supplementary material for: Creating Interactive Data Dashboards for Evidence Syntheses
Source: Cochrane Evid Synth Methods. 2025 Jun 25;3(4):e70035. doi: 10.1002/cesm.70035 (PMC12224945; doi:10.1002/cesm.70035)
Supplement: Supplementary file 1 — Evidence Synthesis Dashboards appendix. [file CESM-3-e70035-s001.docx]

**Appendices**

**Appendix Figure 1. Selected** **screen shots of the abstraction forms in DistillerSR**

**Appendix 1. Defining R Shiny User Interface in Example 2**

**Appendix 2. Defining R Shiny Server Logic**

**Appendix Figure 1. Selected** **screen shots of the abstraction forms in DistillerSR**


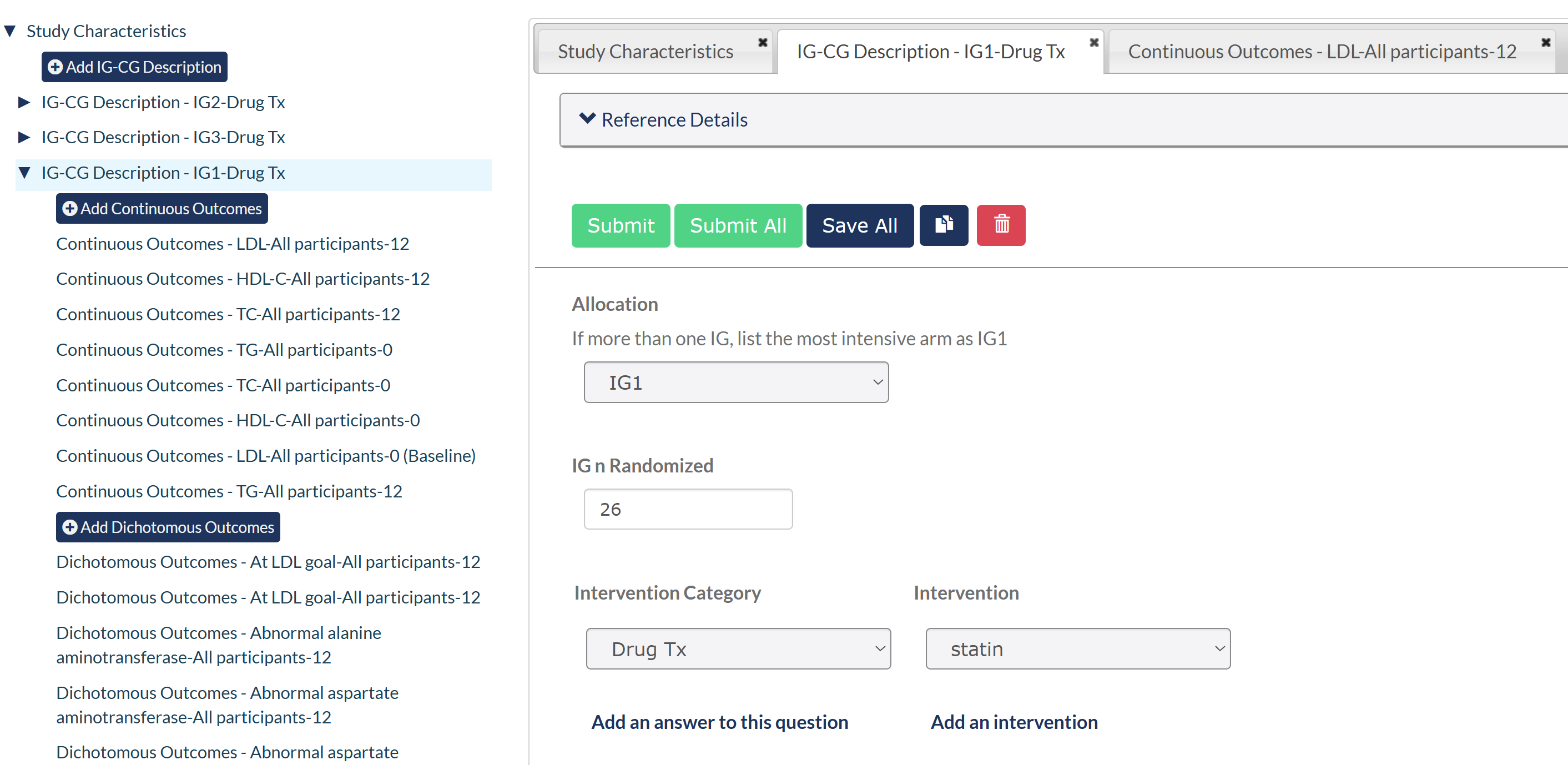

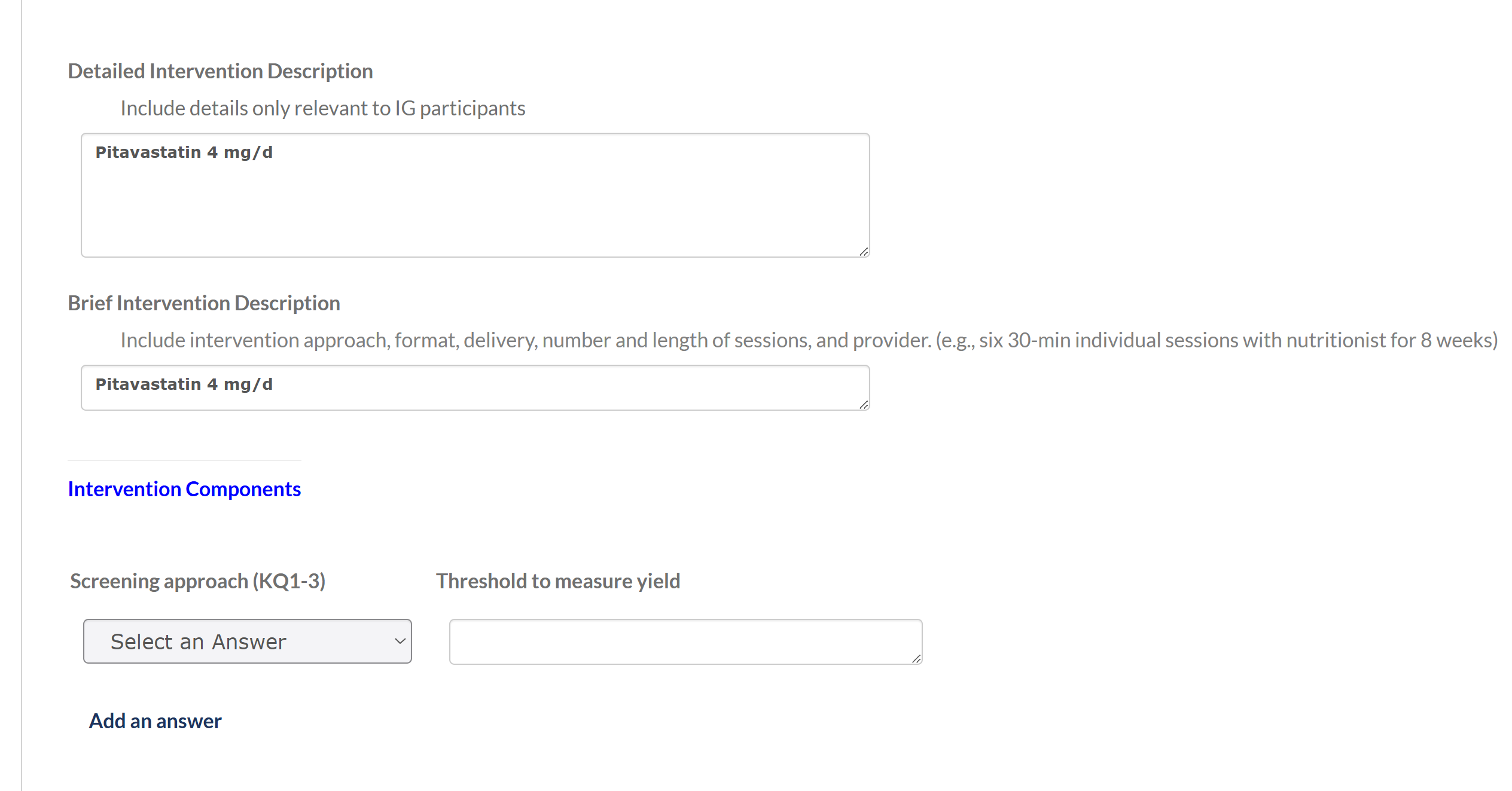

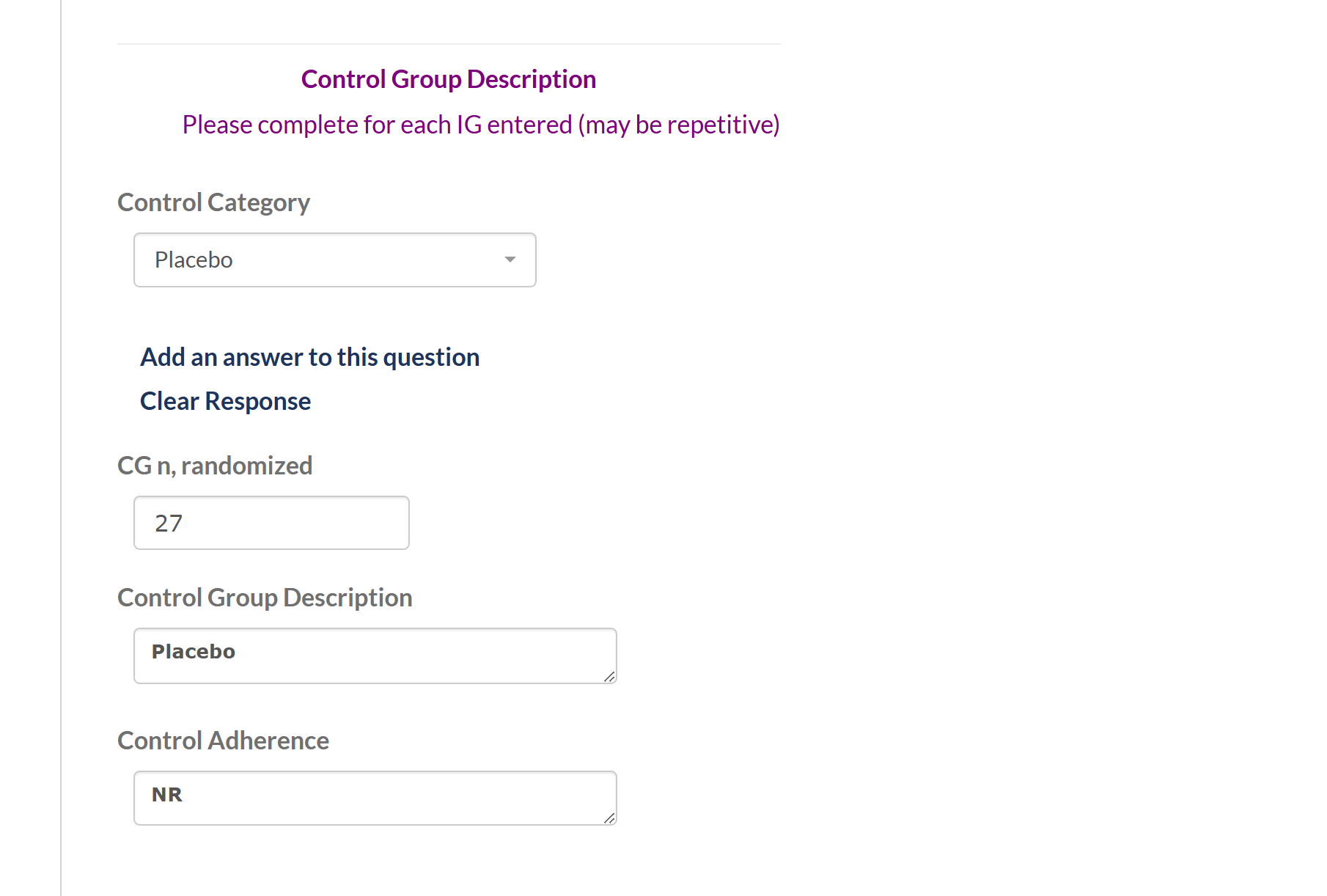

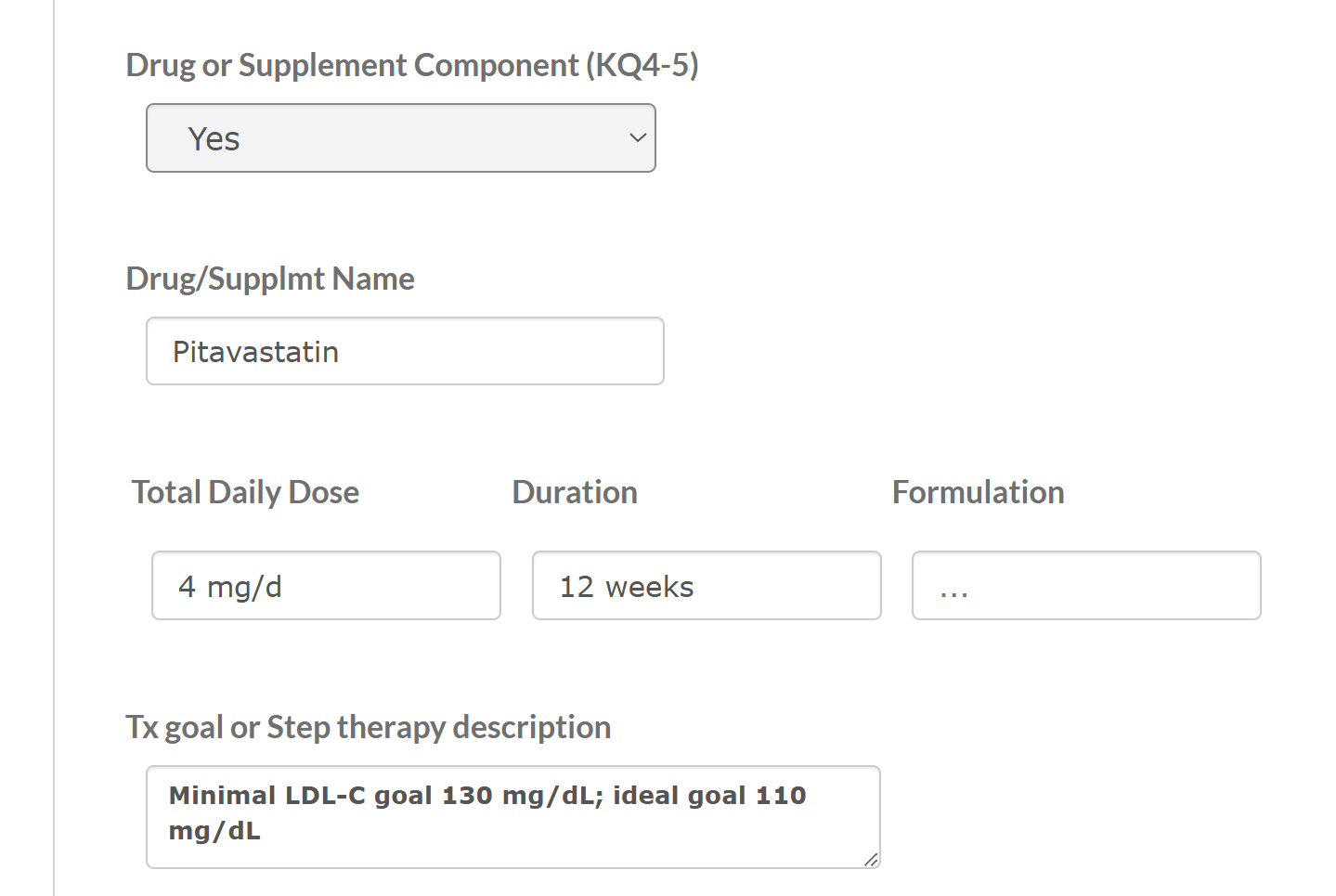

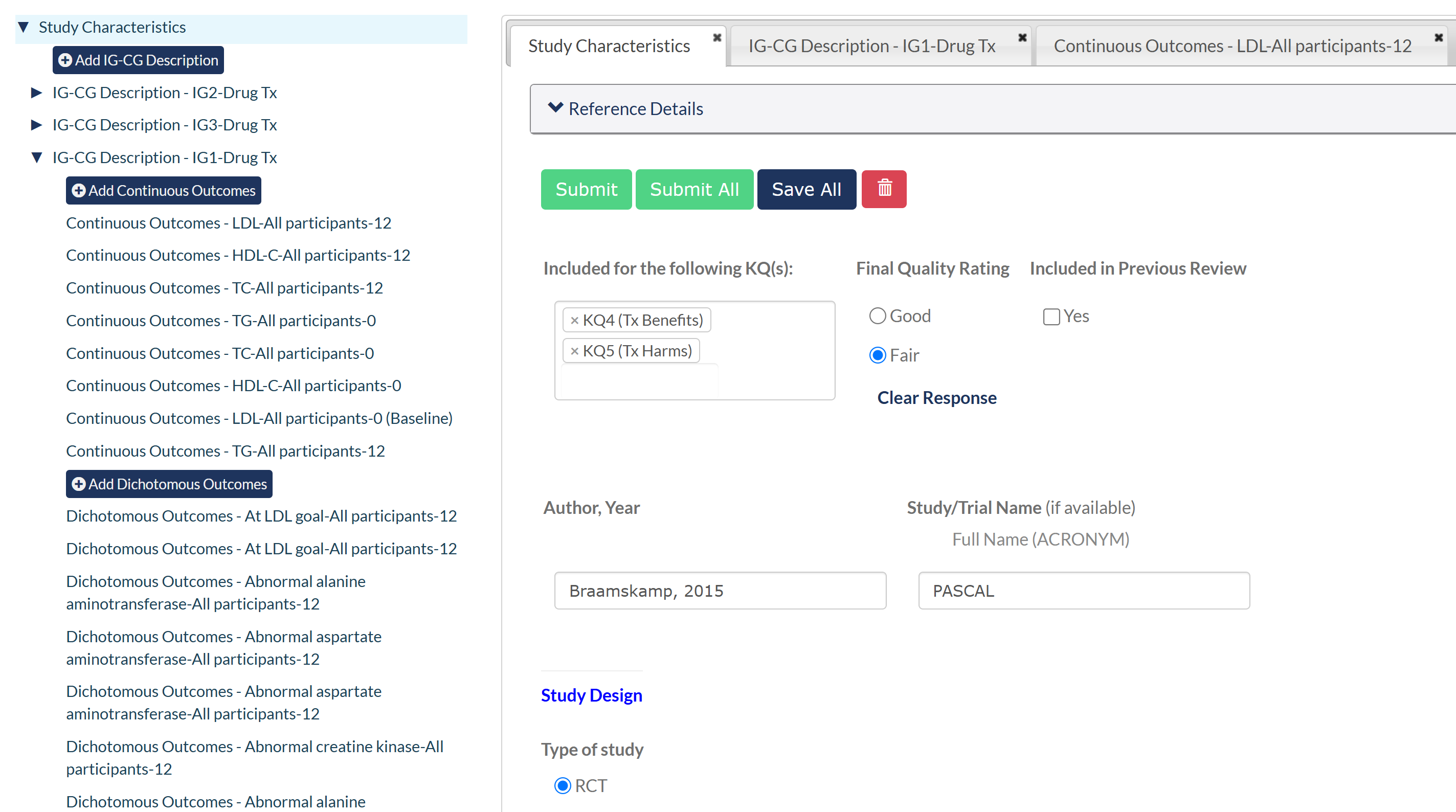

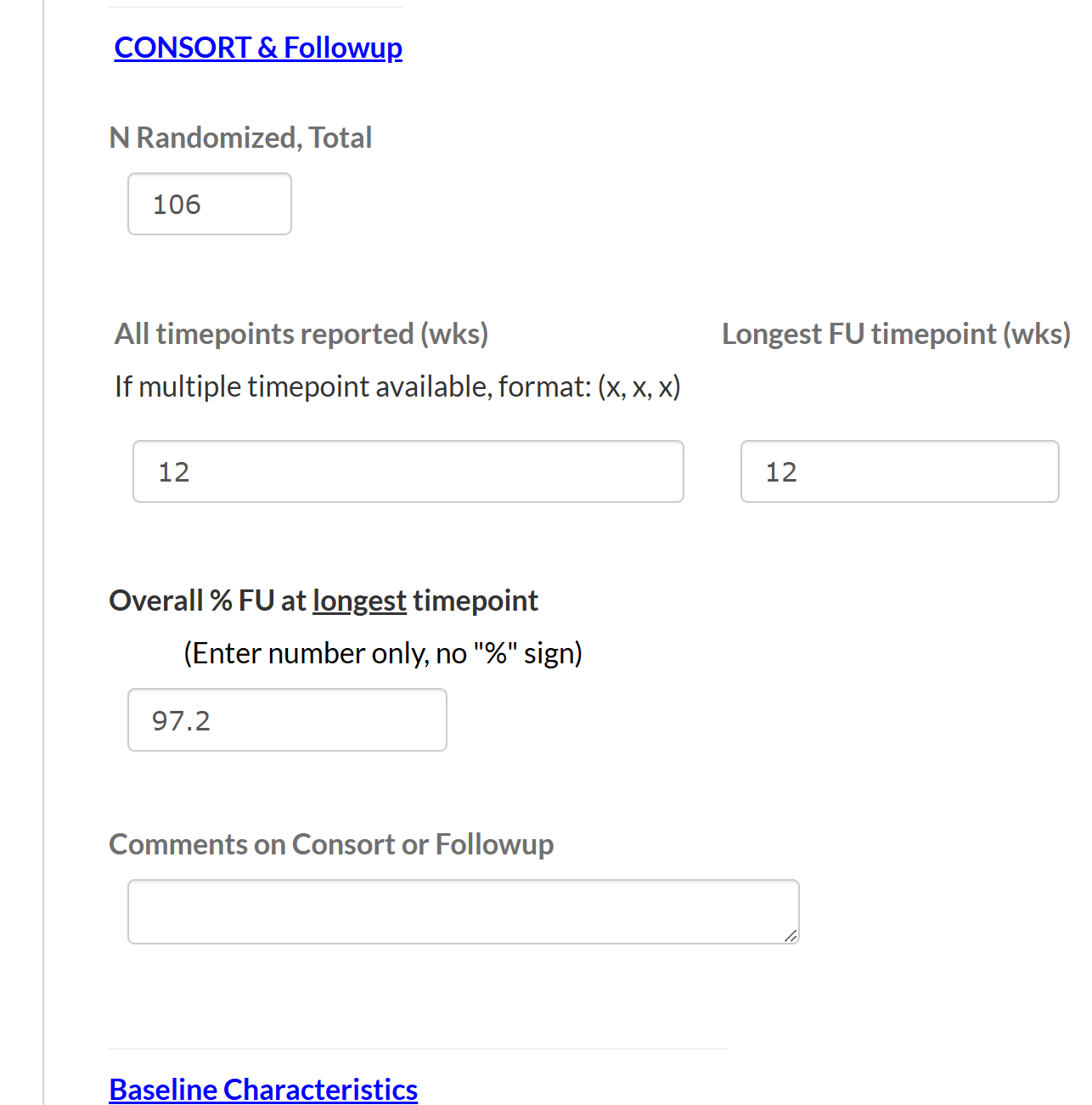


Level 1: Study characteristics

Level 2: Intervention characteristics

Level 3: Results


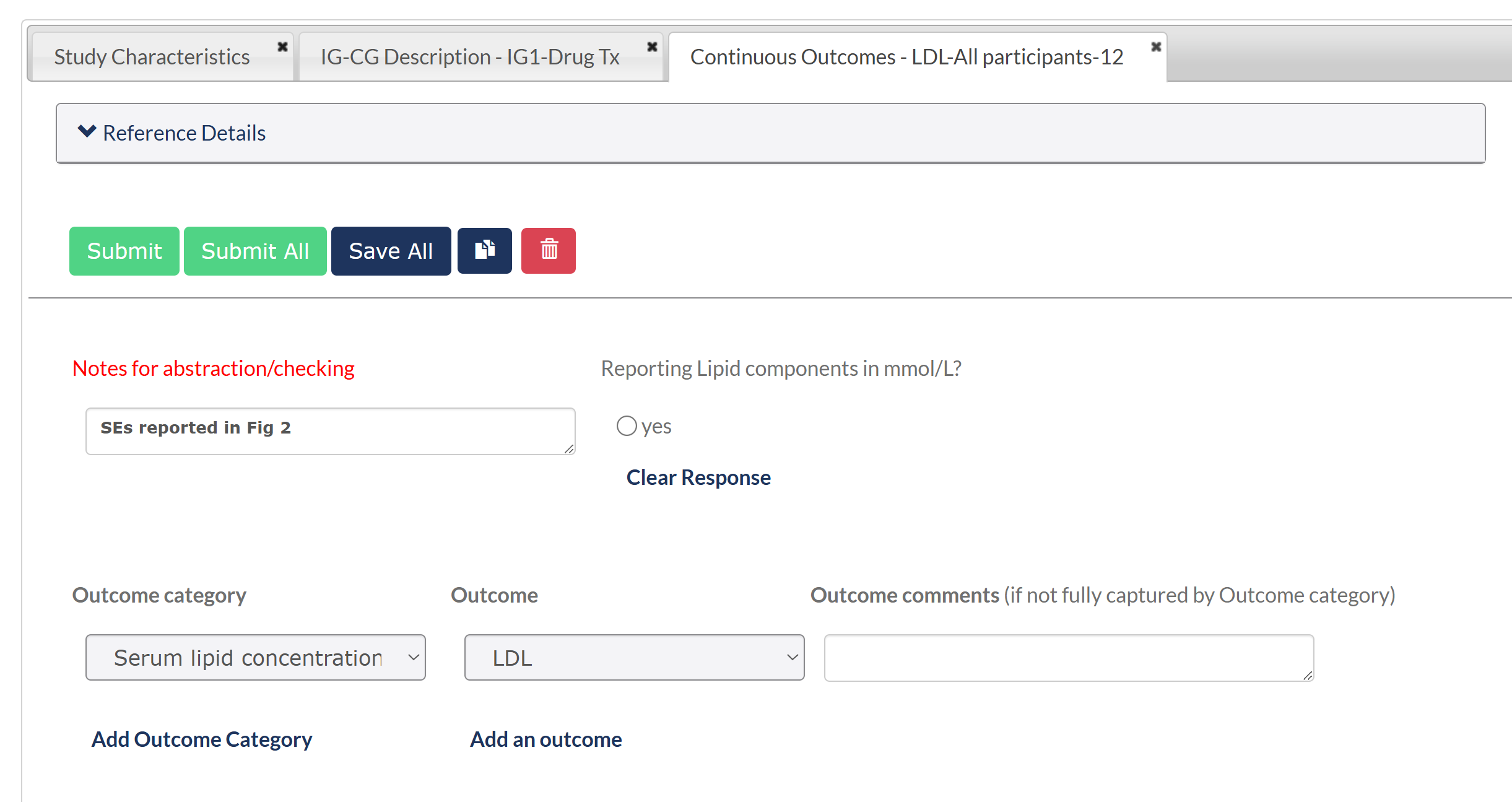

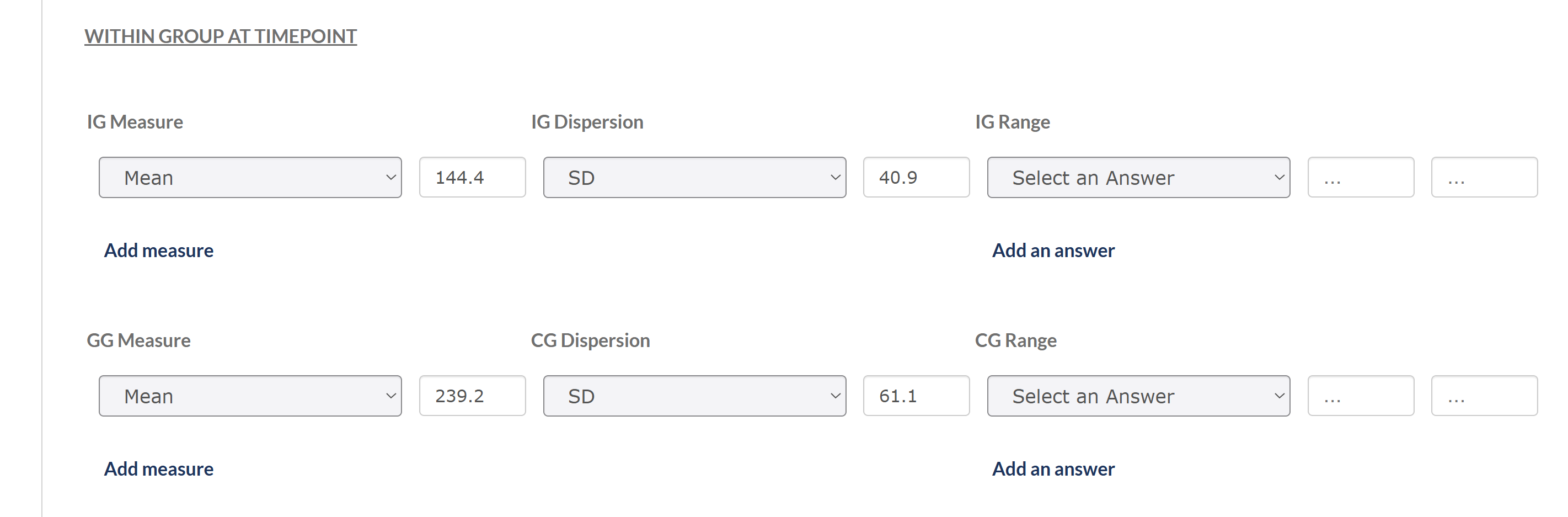

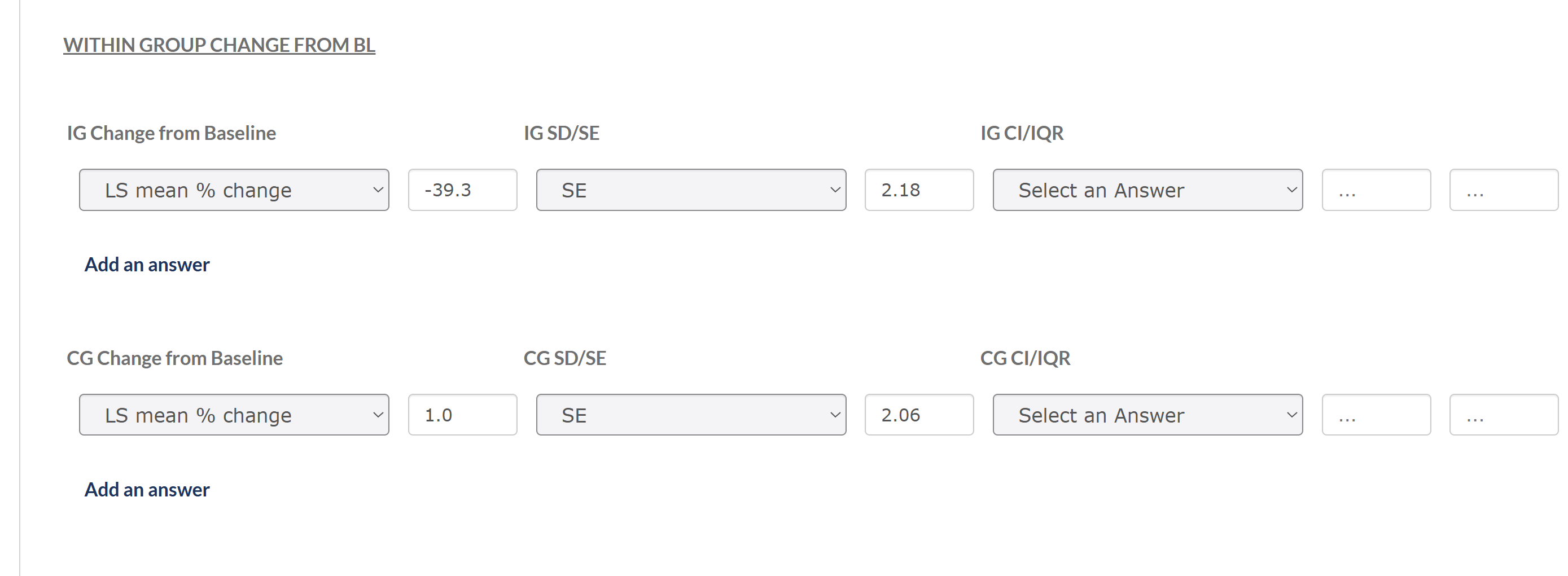


**Appendix 1. Defining R Shiny User Interface in Example 2**

Within the `fluidPage()` function, we first used the `tags$head()` function to embed CSS for customizing the appearance of the UI – such as specifying the font, spacing and borders – and to embed our Google Analytics code for tracking user interactions on the dashboard. Second, we defined the title, step-by-step instructions, and tips using a series of `div()` functions throughout the UI. Third, we use the `plotlyOutput()` function to place the map filter after the instructions for selecting a state. The map filter will allow uses to select states on a map of the U.S. and dynamically filter the data. The interactivity of the map and filters will be built in the server logic section below. Next, we use the fluidRow() function to create new rows to organize and present drop-down filters. Each drop-down filter is defined with `selectizeInput()`. Within `selectizeInput()`, we specified the name of the filter (to reference in the server logic), the text that should appear next to the drop-down box, the filter choices (previously defined in the beginning of the script), and CSS styling to customize the spacing. We defined six `selectizeInput() commands, one for each drop-down filter and included an `actionButton()` to clear filters. Lastly, we used `tabsetPanel()` to create the main section of the dashboard that would be separated into three different tabs. The first `tabPanel()` shows the data table output and two download buttons using `DTOutput()` and `downloadButton()` respectively. The second tab shows the summary statistic table output with `uiOutput()`, and the third tab defines the glossary tab which is a series of HTML text functions to produce a list of definitions for key terms used in the dashboard. While we defined the layout and appearance of the dashboard in the UI, the dynamic outputs that are shown in the UI need to be defined in the server logic.

**Appendix 2. Defining R Shiny Server Logic**

For the interactive map, we first used `plot_geo()` from the *plotly* package to create a monochromatic default map of the U.S. in which states that did not have available data were blank. The map is then saved as an output object using `renderPlotly()` so that it can be referenced in the UI. To enable interactivity, we specify the `event_register()` function to recognize a when a user selects a state on the map (i.e., `plotly_click`). These selections are tracked using reactive values `reactiveVal()`. We defined two reactive variables: `selected_states` and `clicked_state` which store the name of the selected and clicked state(s). We then use the `observeEvent()` function to update the map to highlight the selected states based on the `plotly_click`. If a state is selected, the map is updated to highlight the currently selected states. If no state is selected, the default map is rendered.

To filter the data set based on user selection, we first defined a reactive expression. Within the `reactive()` function, we specified a new data frame based on our preprocessed data. This new `filtered_dataset` object will be dynamically updated when a user modifies any filter inputs in the UI. After specifying a new `filtered_dataset` object, we defined the logic to filter the data for each filter defined in the UI. Our filtering logic starts with a filter for states selected on the map. If one or more states are selected, the data set is filtered to only include those states using the `filter()` function and a regular R expression, `grepl()` to match selected states with the state variable in the data set. Subsequent filters are defined for each filter specified in the UI (e.g., community type, school type, race/ethnicity) and use a combination of `grepl()`, `do.call()`, and `lapply()` to achieve complex filtering logic that can handle multiple responses and partial matches. The `filtered_dataset` is continuously updated as users interact with the filters.

After filtering the data set, the next step in the server logic is to specify how to present the filtered dataset in the dashboard. We used the `renderDataTable()` function from the *DT* package to generate a nicely formatted HTML table of our `filtered_dataset`. Within this function, we loaded our `filtered_dataset` and used conditional logic so that if the `filtered_dataset` contains no rows (i.e., no data matches user filters), an empty table is rendered with the message: “No data matches your filters.” If there is data that matches user’s filters, the `filtered_dataset` is modified to include only key variables and formatted for the table (e.g., capitalized column names, disabling row names, specifying column widths, specifying the number of rows to display per page). Lastly, we use the `observeEvent()` function to provide reset functionality for our filters. When a user clicks on the reset button added in the UI (`input$resetFilters`), all filter selections are cleared and the map is reset to its original state.

To create summary statistic tables, we used the `renderUI()` function to calculate and display a summary statistic table for each variable in the dashboard. Within `renderUI()`, we loaded our `filtered_dataset` and applied conditional logic so that if the `filtered_dataset` contained no data that matched the user’s criteria, a message was displayed: “No data matches your filters.” If the `filtered_dataset` does contain data, it is reformatted to calculate a count and percentage of each category for all variables. We then used separate `div()` functions and CSS code to present the tables in two columns and in a specific order so users could easily view all tables on one page.

The final step in setting up our server logic was to add functionality to the download buttons we specified in the UI. We used the `downloadHandler()` function to generate downloadable Excel files so users could export either the complete data or their filtered data. For the complete data, we used the `write.xlsx()` function to export a cleaned version of the complete data set that was created in beginning of the script. To create the download functionality for the filtered data, we had to load the `filtered_dataset`, clean up some of the column formatting (e.g., removing links), and export using `write.xlsx()`.
